# Supplementary material for: Sufficiency of the BOT-2 short form to screen motor competency in preschool children with strabismus
Source: PLoS One. 2021 Dec 20;16(12):e0261549. doi: 10.1371/journal.pone.0261549 (PMC8687543; doi:10.1371/journal.pone.0261549)
Supplement: S1 Table — (DOCX) [file pone.0261549.s001.docx]

**S1 Table. Subtests (Fine Motor Precision/Fine Motor Integration/Manual Dexterity/Upper-Limb Coordination) and composite (Fine Manual control/Manual Coordination) results of BOT-2 in preschool children with strabismus.**

| **Subjects** | **Strabismus type** | **Stereoacuity**  **(arc sec)** | **Fine Motor Precision** | | **Fine Motor Integration** | | **Fine Manual control** | | | **Manual Dexterity** | | **Upper-Limb Coordination** | | **Manual Coordination** | | |
| --- | --- | --- | --- | --- | --- | --- | --- | --- | --- | --- | --- | --- | --- | --- | --- | --- |
|  |  |  | **ScS** | **DC** | **ScS** | **DC** | **StS** | **%ile** | **DC** | **ScS** | **DC** | **ScS** | **DC** | **StS** | **%ile** | **DC** |
| 1 | ET | 400 | 7 | BA | 7 | BA | 31 | 3% | BA | 6 | BA | 8 | BA | 30 | 2% | WBA |
| 2 | XT+left hypertropia | not detectable | 12 | A | 12 | A | 42 | 21% | A | 8 | BA | 8 | BA | 32 | 4% | BA |
| 3 | ET | not detectable | 18 | A | 15 | A | 53 | 62% | A | 13 | A | 11 | A | 43 | 24% | A |
| 4 | ET | not detectable | 7 | BA | 7 | BA | 31 | 3% | BA | 6 | BA | 7 | BA | 29 | 2% | WBA |
| 5 | ET | not detectable | 24 | AA | 20 | AA | 66 | 95% | AA | 14 | A | 8 | BA | 40 | 16% | BA |
| 6 | ET | not detectable | 9 | BA | 11 | A | 38 | 12% | BA | 4 | WBA | 10 | BA | 30 | 2% | WBA |
| 7 | XT+vertical | not detectable | 26 | WAA | 22 | AA | 71 | 98% | WAA | 18 | A | 15 | A | 53 | 62% | A |
| 8 | ET | not detectable | 16 | A | 16 | A | 52 | 58% | A | 7 | BA | 10 | BA | 34 | 6% | BA |
| 9 | ET | 800 | 16 | A | 13 | A | 48 | 42% | A | 14 | A | 16 | A | 50 | 50% | A |
| 10 | ET | not detectable | 13 | A | 10 | BA | 41 | 18% | A | 4 | WBA | 3 | WBA | 21 | <1% | WBA |
| 11 | XT+vertical | not detectable | 13 | A | 14 | A | 46 | 35% | A | 6 | BA | 14 | A | 37 | 10% | BA |
| 12 | XT | not detectable | 13 | A | 15 | A | 47 | 38% | A | 16 | A | 15 | A | 50 | 50% | A |
| 13 | ET | not detectable | 22 | AA | 17 | A | 60 | 84% | AA | 9 | BA | 10 | BA | 36 | 8% | BA |
| 14 | ET | 400 | 14 | A | 15 | A | 48 | 42% | A | 8 | BA | 10 | BA | 35 | 7% | BA |
| 15 | ET | not detectable | 12 | A | 16 | A | 47 | 38% | A | 18 | A | 17 | A | 54 | 66% | A |
| 16 | ET | not detectable | 24 | AA | 19 | A | 64 | 92% | AA | 13 | A | 14 | A | 46 | 35% | A |
| 17 | ET | 400 | 23 | AA | 21 | AA | 66 | 95% | AA | 13 | A | 5 | WBA | 35 | 7% | BA |
| 18 | ET | not detectable | 20 | AA | 14 | A | 54 | 66% | A | 11 | A | 11 | A | 40 | 16% | BA |
| 19 | XT | not detectable | 16 | A | 11 | A | 46 | 35% | A | 6 | BA | 12 | A | 35 | 7% | BA |
| 20 | ET | 400 | 25 | WAA | 14 | A | 60 | 84% | AA | 6 | BA | 12 | A | 35 | 7% | BA |
| 21 | XT | 400 | 13 | A | 17 | A | 50 | 50% | A | 11 | A | 9 | BA | 37 | 10% | BA |
| 22 | ET | not detectable | 15 | A | 13 | A | 47 | 38% | A | 7 | BA | 9 | BA | 32 | 4% | BA |
| 23 | ET | not detectable | 25 | WAA | 14 | AA | 60 | 84% | AA | 8 | BA | 7 | BA | 31 | 3% | BA |
| 24 | XT | not detectable | 5 | WBA | 2 | WBA | 24 | 1% | WBA | 5 | WBA | 8 | BA | 29 | 2% | WBA |
| 25 | ET | not detectable | 14 | A | 8 | BA | 40 | 16% | BA | 10 | BA | 13 | A | 41 | 18% | A |
| 26 | XT+vertical | 400 | 16 | A | 11 | A | 46 | 35% | A | 7 | BA | 10 | BA | 36 | 8% | BA |
| 27 | Vertical | not detectable | 19 | A | 14 | A | 53 | 62% | A | 17 | A | 10 | BA | 46 | 35% | A |
| 28 | ET | not detectable | 15 | A | 16 | A | 51 | 54% | A | 8 | BA | 7 | BA | 31 | 3% | BA |
| 29 | ET | not detectable | 13 | A | 5 | WBA | 37 | 10% | BA | 7 | A | 5 | WBA | 32 | 4% | BA |
| 30 | XT | 400 | 15 | A | 12 | A | 46 | 35% | A | 11 | A | 8 | BA | 37 | 10% | BA |
| 31 | XT | not detectable | 15 | A | 9 | BA | 43 | 24% | A | 10 | BA | 10 | BA | 38 | 12% | BA |
| 32 | ET | not detectable | 13 | A | 15 | A | 47 | 38% | A | 12 | A | 10 | BA | 41 | 18% | A |
| 33 | ET | not detectable | 21 | AA | 24 | AA | 68 | 96% | AA | 13 | A | 8 | BA | 39 | 14% | BA |
| 34 | ET+Right hypertropia | 400 | 22 | AA | 20 | AA | 65 | 92% | AA | 13 | A | 9 | BA | 40 | 16% | BA |
| 35 | XT | 400 | 17 | A | 16 | A | 53 | 62% | A | 11 | A | 13 | A | 42 | 21% | A |
| 36 | XT+vertical | not detectable | 15 | A | 15 | A | 49 | 46% | A | 18 | A | 13 | A | 50 | 50% | A |
| 37 | ET | not detectable | 11 | A | 12 | A | 42 | 21% | A | 8 | BA | 9 | BA | 36 | 8% | BA |
| 38 | ET | not detectable | 24 | AA | 20 | AA | 69 | 97% | AA | 8 | BA | 5 | WBA | 32 | 4% | BA |
| 39 | ET | not detectable | 16 | A | 12 | A | 47 | 38% | A | 10 | BA | 13 | A | 41 | 18% | A |
| 40 | ET | not detectable | 12 | A | 15 | A | 46 | 35% | A | 11 | A | 10 | BA | 39 | 14% | BA |
|  | | Range | 5－26 |  | 2－24 |  | 24－71 | 1%－98% |  | 4－18 |  | 3－17 |  | 21－54 | 2%－66% |  |
|  |  | Average | 16.15 |  | 13.98 |  | 49.85 | 49.88% |  | 10.13 |  | 10.05 |  | 37.88 | 16.74% |  |
|  |  | SD | 5.31 |  | 4.62 |  | 10.87 | 29.86% |  | 3.92 |  | 3.17 |  | 7.18 | 17.27% |  |
| ET: Esotropia; AC/A: Accommodation convergence/accommodation; XT: Exotropia; ScS: Scale Score; DC: Descriptive Category; StS: Standard Score; WAA: Well-Above Average (Scale Score≥25; Standard Score≥70; %ile Rank≥98); AA: Above Average (24≥Scale Score≥20; 69≥Standard Score≥60; 97≥%ile Rank≥84); A: Average (19≥Scale Score≥11; 59≥Standard Score≥41; 83≥%ile Rank≥18); BA: Below Average (10≥Scale Score≥6; 40≥Standard Score≥31; 17≥%ile Rank≥3); WBA: Well-Below Average (Scale Score≤5; Standard Score≤30; %ile Rank≤2) | | | | | | | | | | | | | | | | |
