# Supplementary material for: Sufficiency of the BOT-2 short form to screen motor competency in preschool children with strabismus
Source: PLoS One. 2021 Dec 20;16(12):e0261549. doi: 10.1371/journal.pone.0261549 (PMC8687543; doi:10.1371/journal.pone.0261549)
Supplement: S2 Table — (DOCX) [file pone.0261549.s002.docx]

**S2 Table. Subtests (Bilateral Coordination/Balance/Running Speed and Agility/Strength), composite (Body Coordination/ Strength and Agility), and Total Motor Composite of Complete-form/Short-form results of BOT-2 in preschool children with strabismus.**

| **Subjects** | **Bilateral Coordination** | | **Balance** | | **Body Coordination** | | | **Running Speed and Agility** | | **Strength** | | **Strength and Agility** | | | **Total Motor Composite: Complete form** | | | **Total Motor Composite: Short form** | | |
| --- | --- | --- | --- | --- | --- | --- | --- | --- | --- | --- | --- | --- | --- | --- | --- | --- | --- | --- | --- | --- |
|  | **ScS** | **DC** | **ScS** | **DC** | **StS** | **%ile** | **DC** | **ScS** | **DC** | **ScS** | **DC** | **StS** | **%ile** | **DC** | **StS** | **%ile** | **DC** | **StS** | **%ile** | **DC** |
| 1 | 9 | BA | 4 | WBA | 32 | 4% | BA | 12 | A | 18 | A | 49 | 46% | A | 33 | 5% | BA | 43 | 24% | A |
| 2 | 12 | A | 23 | AA | 56 | 73% | A | 17 | A | 24 | AA | 63 | 90% | AA | 47 | 38% | A | 51 | 54% | A |
| 3 | 19 | A | 16 | A | 56 | 73% | A | 16 | A | 13 | A | 48 | 42% | A | 49 | 46% | A | 64 | 92% | AA |
| 4 | 11 | A | 5 | WBA | 34 | 6% | BA | 12 | A | 14 | A | 44 | 27% | A | 32 | 4% | BA | 56 | 73% | A |
| 5 | 20 | AA | 22 | AA | 63 | 90% | AA | 21 | AA | 28 | WAA | 70 | 98% | WAA | 63 | 90% | AA | 58 | 79% | A |
| 6 | 10 | BA | 10 | BA | 38 | 12% | BA | 11 | A | 9 | BA | 38 | 12% | BA | 34 | 6% | BA | 41 | 18% | A |
| 7 | 20 | AA | 27 | WAA | 72 | 99% | WAA | 22 | AA | 24 | AA | 68 | 96% | AA | 74 | 99% | WAA | 64 | 92% | AA |
| 8 | 18 | A | 12 | A | 50 | 50% | A | 15 | A | 14 | A | 48 | 42% | A | 44 | 27% | A | 58 | 79% | A |
| 9 | 18 | A | 20 | AA | 59 | 82% | A | 14 | A | 23 | AA | 60 | 84% | AA | 56 | 73% | A | 57 | 76% | A |
| 10 | 5 | WBA | 7 | BA | 32 | 4% | BA | 15 | A | 18 | A | 52 | 58% | A | 34 | 6% | BA | 43 | 24% | A |
| 11 | 11 | A | 10 | BA | 39 | 14% | BA | 7 | BA | 18 | A | 42 | 21% | A | 38 | 12% | BA | 66 | 95% | AA |
| 12 | 12 | A | 16 | A | 47 | 38% | A | 27 | WAA | 26 | WAA | 75 | 99% | WAA | 56 | 73% | A | 46 | 35% | A |
| 13 | 18 | A | 16 | A | 54 | 66% | A | 21 | AA | 23 | AA | 67 | 96% | AA | 55 | 69% | A | 43 | 24% | A |
| 14 | 11 | A | 16 | A | 46 | 35% | A | 15 | A | 23 | AA | 60 | 84% | AA | 46 | 35% | A | 46 | 35% | A |
| 15 | 11 | A | 17 | A | 46 | 35% | A | 20 | AA | 23 | AA | 64 | 92% | AA | 53 | 62% | A | 49 | 46% | A |
| 16 | 10 | BA | 10 | BA | 38 | 12% | BA | 15 | A | 19 | A | 54 | 66% | A | 50 | 50% | A | 52 | 58% | A |
| 17 | 17 | A | 18 | A | 56 | 73% | A | 17 | A | 25 | WAA | 65 | 93% | AA | 57 | 76% | A | 56 | 73% | A |
| 18 | 12 | A | 14 | A | 44 | 27% | A | 21 | AA | 24 | AA | 68 | 96% | AA | 52 | 58% | A | 44 | 27% | A |
| 19 | 11 | A | 9 | BA | 38 | 12% | BA | 16 | A | 18 | A | 54 | 66% | A | 41 | 18% | A | 73 | 99% | WAA |
| 20 | 12 | A | 23 | AA | 56 | 73% | A | 17 | A | 23 | AA | 62 | 89% | AA | 54 | 66% | AA | 61 | 86% | AA |
| 21 | 3 | WBA | 13 | A | 34 | 6% | BA | 13 | A | 18 | A | 51 | 54% | A | 41 | 18% | A | 49 | 46% | A |
| 22 | 3 | WBA | 15 | A | 36 | 8% | BA | 22 | AA | 24 | AA | 68 | 96% | AA | 44 | 27% | A | 45 | 31% | A |
| 23 | 13 | A | 14 | A | 46 | 35% | A | 15 | A | 17 | A | 51 | 54% | A | 45 | 31% | A | 44 | 27% | A |
| 24 | 3 | WBA | 3 | WBA | 24 | 1% | WBA | 10 | BA | 11 | A | 39 | 14% | BA | 25 | 1% | WAB | 46 | 35% | A |
| 25 | 16 | A | 17 | A | 53 | 62% | A | 20 | AA | 28 | WAA | 69 | 97% | AA | 50 | 50% | A | 60 | 84% | AA |
| 26 | 9 | BA | 16 | A | 43 | 24% | A | 15 | A | 17 | A | 52 | 58% | A | 42 | 21% | A | 53 | 62% | A |
| 27 | 8 | BA | 16 | A | 42 | 21% | A | 20 | AA | 23 | AA | 66 | 95% | AA | 52 | 58% | A | 65 | 93% | AA |
| 28 | 13 | A | 6 | BA | 37 | 10% | BA | 12 | A | 15 | A | 45 | 31% | A | 38 | 12% | BA | 51 | 54% | A |
| 29 | 12 | A | 11 | A | 41 | 18% | A | 16 | A | 19 | A | 56 | 73% | A | 39 | 14% | BA | 51 | 54% | A |
| 30 | 5 | WBA | 8 | BA | 32 | 4% | BA | 14 | A | 16 | A | 50 | 50% | A | 38 | 12% | BA | 52 | 58% | A |
| 31 | 5 | WBA | 22 | AA | 46 | 35% | A | 22 | AA | 28 | WAA | 74 | 99% | WAA | 50 | 50% | A | 33 | 5% | BA |
| 32 | 16 | A | 15 | A | 51 | 54% | A | 19 | A | 17 | A | 56 | 73% | A | 48 | 42% | A | 57 | 76% | A |
| 33 | 13 | A | 8 | BA | 39 | 14% | BA | 18 | A | 26 | WAA | 67 | 96% | AA | 54 | 66% | A | 59 | 82% | A |
| 34 | 13 | A | 16 | A | 47 | 38% | A | 16 | A | 22 | AA | 60 | 84% | AA | 54 | 66% | A | 61 | 86% | AA |
| 35 | 13 | A | 11 | A | 42 | 21% | A | 19 | A | 26 | WAA | 68 | 96% | AA | 51 | 54% | A | 64 | 92% | AA |
| 36 | 7 | BA | 16 | A | 41 | 18% | A | 19 | A | 22 | AA | 63 | 90% | AA | 50 | 50% | A | 54 | 66% | A |
| 37 | 16 | A | 14 | A | 48 | 42% | A | 16 | A | 21 | AA | 59 | 82% | A | 44 | 27% | A | 32 | 4% | BA |
| 38 | 14 | A | 16 | A | 49 | 46% | A | 19 | A | 19 | A | 58 | 79% | A | 52 | 58% | A | 47 | 38% | A |
| 39 | 10 | BA | 14 | A | 42 | 21% | A | 16 | A | 22 | AA | 60 | 84% | AA | 46 | 35% | A | 66 | 95% | AA |
| 40 | 8 | BA | 18 | A | 44 | 27% | A | 20 | AA | 22 | AA | 64 | 92% | AA | 47 | 38% | A | 37 | 10% | BA |
| Range | 3－20 |  | 3－27 |  | 24－72 | 1%－99% |  | 7－27 |  | 9－28 |  | 38－75 | 12%－99% |  | 25－74 | 1%－99% |  | 32－73 | 4%－99% |  |
| Average | 11.68 |  | 14.1 |  | 44.82 | 34.58% |  | 16.8 |  | 20.5 |  | 58.18 | 72.35% |  | 46.95 | 41.08% |  | 52.43 | 57.18% |  |
| SD | 4.65 |  | 5.47 |  | 9.6 | 27.04% |  | 3.92 |  | 4.71 |  | 9.58 | 26.31% |  | 9.21 | 25.68% |  | 9.53 | 28.66% |  |
| StS: Standard Score; DC: Descriptive Category; WAA: Well-Above Average (Scale Score≥25; Standard Score≥70; %ile Rank≥98); AA: Above Average (24≥Scale Score≥20; 69≥Standard Score≥60; 97≥%ile Rank≥84); A: Average (19≥Scale Score≥11; 59≥Standard Score≥41; 83≥%ile Rank≥18); BA: Below Average (10≥Scale Score≥6; 40≥Standard Score≥31; 17≥%ile Rank≥3); WBA: Well-Below Average (Scale Score≤5; Standard Score≤30; %ile Rank≤2) | | | | | | | | | | | | | | | | | | | | |
