# Supplementary material for: Sufficiency of the BOT-2 short form to screen motor competency in preschool children with strabismus
Source: PLoS One. 2021 Dec 20;16(12):e0261549. doi: 10.1371/journal.pone.0261549 (PMC8687543; doi:10.1371/journal.pone.0261549)
Supplement: S3 Table — (DOCX) [file pone.0261549.s003.docx]

**S3 Table. Results of the BOT-2 between undetectable and detectable stereopsis / with ADHD tendency and without ADHD tendency judged by the Disruptive Behavior Rating Scales in preschool children with strabismus.**

| **n=40** | **Undetectable stereopsis**  **(n=30)** | **Detectable stereopsis**  **(n=10)** | **Between group different**  **P value**  **(95% CI)** | **Effect size**  **(95% CI)** | **With ADHD tendency**  **(n=21)** | **Without ADHD tendency**  **(n=19)** | **Between group different**  **P value**  **(95% CI)** | **Effect size (95% CI)** |
| --- | --- | --- | --- | --- | --- | --- | --- | --- |
|  | **Scale Score (Mean/SD)** | |  |  | **Scale score (Mean/SD)** | |  |  |
| Fine Motor Precision | 15.93±5.38 | 16.8±5.33 | 0.661  (-4.836, 3.102) | -0.162  (-0.879, 0.555) | 15.29±5.29 | 17.11±5.31 | 0.285  (-1.579, 5.218) | -0.343  (-0.969, 0.282) |
| Fine Motor Integration | 13.77±4.8 | 14.6±4.2 | 0.627  (-4.279, 2.613) | -0.178  (-0.895, 0.539) | 13.14±4.43 | 14.89±4.76 | 0.235  (-1.190, 4.694) | -0.381  (-1.008, 0.245) |
| Fine Manual Control | 49.37±11.15 | 51.3±10.4 | 0.632  (-10.050, 6.183) | -0.176  (-0.893, 0.541) | 47.81±10.15 | 52.11±11.46 | 0.216  (-2.621, 11.213) | -0.399  (-1.025, 0.228) |
| Manual Dexterity | 10.17±4.23 | 10±3.03 | 0.909  (-2.771, 3.104) | 0.043  (-0.673, 0.759) | 10.14±4.17 | 10.11±3.74 | 0.976  (-2.585, 2.510) | 0.008  (-0.613, 0.628) |
| Upper-Limb Coordination | 10.07±3.26 | 10±3.06 | 0.955  (-2.307, 2.440) | 0.022  (-0.694, 0.738) | 10.48±3.2 | 9.58±3.15 | 0.378  (-2.934, 1.140) | 0.283  (-0.340, 0.907) |
| Manual Coordination | 37.93±7.77 | 37.7±5.38 | 0.930  (-5.143, 5.609) | 0.032  (-0.684, 0.747) | 38.48±7.64 | 37.21±6.78 | 0.584  (-5.909, 3.378) | 0.175  (-0.447, 0.797) |
| Bilateral Coordination | 11.9±4.67 | 11±4.74 | 0.603  (-2.573, 4.373) | 0.192  (-0.525, 0.909) | 11.76±5.03 | 11.58±4.33 | 0.903  (-3.205, 2.839) | 0.038  (-0.582, 0.659) |
| Balance | 13.97±5.49 | 14.5±5.66 | 0.793  (-4.624, 3.557) | -0.096  (-0.812, 0.620) | 12.71±4.93 | 15.63±5.75 | 0.092  (-0.501, 6.336) | -0.547  (-1.180, 0.085) |
| Body Coordination | 44.87±9.61 | 44.7±10.08 | 0.963  (-7.022, 7.355) | 0.018  (-0.698, 0.733) | 43.29±9.63 | 46.53±9.52 | 0.292  (-2.901, 9.382) | -0.338  (-0.963, 0.287) |
| Running Speed and Agility | 17.33±34.25 | 15.2±2.1 | 0.045*  (0.053, 4.214) | 0.071  (-0.645, 0.787) | 15.9±3.94 | 17.79±3.75 | 0.130  (-0.582, 4.352) | -0.491  (-1.121, 0.139) |
| Strength | 20.3±5.07 | 21.1±3.54 | 0.648  (-4.315, 2.715) | -0.168  (-0.885, 0.548) | 18.67±4.79 | 22.53±3.78 | 0.008*  (1.079, 6.641) | -0.889*  (-1.540, -0.239) |
| Strength and Agility | 58.33±10.45 | 57.7±6.71 | 0.859  (-6.534, 7.801) | 0.065  (-0.651, 0.781) | 54.9±9.56 | 61.79±8.42 | 0.021*  (1.093, 12.677) | -0.762*  (-1.405, -0.120) |
| Total Motor Composite: Complete form | 46.87±9.6 | 47.2±8.39 | 0.923  (-7.227, 6.561) | -0.035  (-0.751, 0.680) | 44.86±9.05 | 49.26±9.05 | 0.132  (-1.395, 10.207) | -0.486  (-1.116, 0.144) |
| Total Motor Composite: Short form | 53±9.66 | 50.6±9.38 | 0.492  (-4.658, 9.525) | 0.250  (-0.468, 0.968) | 52±10.65 | 52.89±8.39 | 0.771  (-5.283, 70.76) | -0.092  (-0.713, 0.529) |
| *: p-value<0.05 | | | | | | | | |
