# Supplementary material for: Sufficiency of the BOT-2 short form to screen motor competency in preschool children with strabismus
Source: PLoS One. 2021 Dec 20;16(12):e0261549. doi: 10.1371/journal.pone.0261549 (PMC8687543; doi:10.1371/journal.pone.0261549)
Supplement: S4 Table — (DOCX) [file pone.0261549.s004.docx]

**S4 Table. Correlation between BOT-2 SF items and corresponding domain scores from the BOT-2 CF.**

| **Domain** | **BOT-2 SF items** | **Pearson product-moment correlation coefficient** |
| --- | --- | --- |
| Fine motor precision | 3. Drawing lines through paths—crooked | 0.639* |
|  | 6. Folding paper | 0.736* |
| Fine motor integration | 2. Copying a square | 0.743* |
|  | 7. Copying a star | 0.676* |
| Manual dexterity | 2. Transferring coins | 0.593* |
| Bilateral coordination | 3. Jumping in place—same sides synchronized | 0.603* |
|  | 6. Tapping feet and fingers—same sides synchronized | 0.681^ǂ^* |
| Balance | 2. Walking forward on a line | NA (Because the point score was 4 in all subjects) |
|  | 7. Standing on one leg on a balance beam—eyes open | 0.669* |
| Running speed & agility | 3. One-legged stationary hop | 0.719* |
| Upper-limb coordination | 1. Dropping and catching a ball—both hands | 0.393^+^ |
|  | 6. Dribbling a ball—alternating hands | 0.373^+^ |
| Strength | 2. Push-ups | 0.653* |
|  | 3. Sit-ups | 0.712* |
| *: p<0.01; ^+^: p<0.05. | | |
