# Supplementary material for: Sufficiency of the BOT-2 short form to screen motor competency in preschool children with strabismus
Source: PLoS One. 2021 Dec 20;16(12):e0261549. doi: 10.1371/journal.pone.0261549 (PMC8687543; doi:10.1371/journal.pone.0261549)
Supplement: Attachment — Submitted filename: PONE-D-21-21187_PLOS ONE.docx [file pone.0261549.s005.docx]

“Sufficiency of the BOT-2 short form to screen motor competency in preschool children with strabismus”

**General Comments**

The purpose of this study was to examine the motor competency in preschool children with Strabismus and to explore the BOT-2 SF to determine if it is accurate in identifying those at risk of MC. While this paper shows the importance for preschool children with Strabismus and using the BOT-2 CF and SF, I believe this paper would benefit from another thorough read through. Most of the paper should be re-organized with missing information in the various sections. My general concerns are outlined below, followed by comments for each section outlined in the paper.

1. Check grammar throughout the paper.
2. Re-organizing the introduction and discussion to help with clarity to readers.
3. Check out the methods section and include important information that is mentioned below.

**Abstract**

I would recommend using different keywords that are not in your title.

Line 31: Use the acronym CF instead of writing out complete form.

**Introduction**

Authors define Strabismus which is great, however, I would recommend going a little further in the type of diagnosis. What does Strabismus entail? Are there different forms of it? In the second paragraph authors mention previous work and that much work explores single aspects. I would like to know why it’s important to explore both gross and fine at the same time as authors mention in line 61. I think that would be an important statement to add. In paragraph three authors speak about the prevalence of ADHD in those with Strabismus. I believe this is worth mentioning where you talk about Strabismus. I would recommend cleaning up the introduction and make it more organized for the reader.

Line 48: Check grammar starting with “In preschool children….”

Line 59: tend, not tended

Line 104: Add comma after the word “after”.

**Methods**

Participants:

How were participants informed of purpose and procedures? Through email? Face to face?

Procedure:

Need more in the procedure section about administering the BOT-2 CF. Where was it administered, how, and the scoring? April 2018 to June 2020 was this just recruitment or when the whole study took place? If it was a two-year study, there needs to be more in the procedures.

Line 147-149: why is the DBRS questionnaire important here?

Results:

I would recommend putting the percentages near each classification, so it makes it easier for the reader.

Lines 197-198: Why are these results important? This is not part of your purpose statement.

**Discussion**

Authors mention esotropia in the third paragraph, however, it seems a bit unclear what Esotropia is because it has not been mentioned up until this point. If it is a necessary component of Strabismus then I would recommend putting this into your introduction.

In the 6^th^ paragraph starting with line 278-292 authors talk about the finding of the DBRS questionnaire. If this is something that will be used in the study, it needs to be mentioned in the methods and the questionnaire should be described. Also, this should be part of the research question/purpose.

In paragraph 8, authors speak about person’s correlations and the results. Were correlations used to analyze these data? If so, this needs to be mentioned in the data analysis section as well.
