# Supplementary material for: Sufficiency of the BOT-2 short form to screen motor competency in preschool children with strabismus
Source: PLoS One. 2021 Dec 20;16(12):e0261549. doi: 10.1371/journal.pone.0261549 (PMC8687543; doi:10.1371/journal.pone.0261549)
Supplement: Attachment — Submitted filename: Response to Reviewers20211020.docx [file pone.0261549.s006.docx]

**Point-by-point responses to the reviewer’s comments on the manuscript**

“Sufficiency of the BOT-2 short form to screen motor competency in preschool children with strabismus”

The authors would like to thank the reviewers for their thoughtful review of our manuscript and for providing us with comments and suggestions to improve its quality. Point-by-point responses to all comments are provided below.

**General Comments**

The purpose of this study was to examine the motor competency in preschool children with Strabismus and to explore the BOT-2 SF to determine if it is accurate in identifying those at risk of MC. While this paper shows the importance for preschool children with Strabismus and using the BOT-2 CF and SF, I believe this paper would benefit from another thorough read through. Most of the paper should be re-organized with missing information in the various sections. My general concerns are outlined below, followed by comments for each section outlined in the paper.

1. Check grammar throughout the paper.
2. Re-organizing the introduction and discussion to help with clarity to readers.
3. Check out the methods section and include important information that is mentioned below.

**Reply:**

1. This revised manuscript has undergone English proofreading and editing by two English editing experts. Please see the attached supporting document.
2. We have rewritten the introduction to clarify the study aims and to ensure that the manuscript is clear of any ambiguities.
3. Thank you for the suggestion. We have amended the methods section accordingly.

**Abstract**

**Reviewer comments:**

I would recommend using different keywords that are not in your title.

**Reply:**

Thank you for the suggestion. We have rewritten the keywords (Page 3, lines 41): *“Keywords: Preschool, Children, Strabismus, Motor skills, Diagnosis.”*

**Reviewer comments:**

Line 31: Use the acronym CF instead of writing out complete form.

**Reply:**

Thank you for pointing out our mistake. This has been corrected (Page 2, line 30): *“The BOT-2 CF was...”*

**Introduction**

**Reviewer comments:**

Authors define Strabismus which is great, however, I would recommend going a little further in the type of diagnosis. What does Strabismus entail? Are there different forms of it?

**Reply:**

Thank you for the suggestion. We have rewritten the Introduction to clearly state the strabismus (Page 4, lines 43−49): *“Strabismus is defined as a deviation of the eye from perfect ocular alignment and can be further divided into esotropia and exotropia, or less commonly, hypertropia and hypotropia (1). It is one of the most common visual development disorders with a reported global prevalence of 1.93% (95% CI=1.64%−2.21%) (2). For preschool-aged children, the prevalence of strabismus ranges from 1.5% to 3.8% (3-5). Preschool children with strabismus have abnormal stereopsis (6) and are at greater risk of amblyopia (7), which is associated with abnormal binocular vision (8).”*

**Reviewer comments:**

In the second paragraph authors mention previous work and that much work explores single aspects. I would like to know why it’s important to explore both gross and fine at the same time as authors mention in line 61. I think that would be an important statement to add.

**Reply:**

Thank you for the suggestion. This has been amended on (Page 4, lines 53－61): *“Despite development of motor competency at preschool age being critical for the overall development of individuals, information regarding the motor competency of preschool children with strabismus is limited. Motor competency is multidimensional, ranging from simple to combined to complex, which are all inter-related, and may be further divided into gross motor competency and fine motor competency in standardized assessments (17, 18). However, previous studies on children with strabismus tend to focus more on a single aspect, either gross motor competency (10, 11, 13, 16), or fine motor competency (6, 12, 15), rather than complete motor competency (14, 19) (Table 1). Therefore, it is necessary to investigate the complete motor competency of preschool children with strabismus.”*

**Reviewer comments:**

In paragraph three authors speak about the prevalence of ADHD in those with Strabismus. I believe this is worth mentioning where you talk about Strabismus. I would recommend cleaning up the introduction and make it more organized for the reader.

**Reply:**

Thank you for the suggestion. This has been amended on (Page 8−9, lines 79−82): “*Considering the relevance of childhood strabismus to ADHD-related symptoms, whether the BOT-2 SF is effective in testing the motor competency of preschool children with strabismus is worthy of further discussion.”*

**Reviewer comments:**

Line 48: Check grammar starting with “In preschool children….”

**Reply:**

Thank you for pointing out our mistake. Corrections have been made to the following pages: (Page 4, line 46－49): *“For preschool-aged children, the prevalence of strabismus ranges from 1.5% to 3.8% (3-5). Preschool children with strabismus have abnormal stereopsis (6) and are at greater risk of amblyopia (7), which is associated with abnormal binocular vision (8).”*

**Reviewer comments:**

Line 59: tend, not tended

**Reply:**

Thank you for pointing out our mistake. Corrections have been made to the following pages: (Page 4, line 58):*“*… *However, previous studies on children with strabismus tend to focus more”*

**Reviewer comments:**

Line 104: Add comma after the word “after”.

**Reply:**

Thank you for pointing out our mistake. Corrections have been made to the following pages: (Page 10, line 102):*“* *After, preschool children with strabismus…”*

**Methods**

Participants:

**Reviewer comments:**

How were participants informed of purpose and procedures? Through email? Face to face?

**Reply:**

Thank you for the suggestion. This has been amended on (Page 10, lines 102−105): “*After, preschool children with strabismus and their parents were informed about the purpose and procedure of the study face-to-face. Verbal and written assent and written informed consent were obtained from the children and the guardians, respectively.”*

Procedure:

**Reviewer comments:**

Need more in the procedure section about administering the BOT-2 CF. Where was it administered, how, and the scoring?

**Reply:**

Thank you for the suggestion. This has been amended on (Page 10−11, lines 115−133): *” …* *divided into a “Fine Motor Precision” subtest (7 items, score range=0−41 points) and “Fine Motor Integration” subtest (8 items, score range=0−40 points); the “Manual Coordination” composite which is divided into a “Manual Dexterity” subtest (5 items, score range=0−45 points) and “Upper-Limb Coordination” subtest (7 items, score range=0−39 points); the “Body Coordination” composite which is divided into a “Bilateral Coordination” subtest (7 items, score range=0−24 points) and “Balance” subtest (9 items, score range=0−37 points), and the “Strength and Agility” composite which is divided into a “Running Speed and Agility” subtest (5 items, score range=0−52 points) and “Strength” subtest (5 items, score range=0−42 points). Each raw score on a subtest was converted to a point score to allow performance to be measured on a graded scale. The individual item points were summed to derive a subtest point score. The subtest point scores for eight subtests were summed to achieve a “Total Motor Composite”. The “Total Motor Composite” comprising the above motor-area composites provides a measure of the child’s overall motor proficiency. The subtest point scores were used to derive a scale score. Scale scores describe comparability to a normative sample of examinees of the same age and have a mean of 15 with a standard deviation of 5. Additionally, scale and standard scores for subtests and composites can be represented in the form of descriptive categories ranging from well below average to well above average (20).”*; (Page 12−13, lines 166−172):*” BOT-2 CF testing was performed on an individual basis in quiet locations at the Pediatric Physical Therapy (PT) Unit of the Chang Gung Medical Foundation, Linkou Chang Gung Memorial Hospital. All participants were tested by the examiner using the standardized procedures for administration specified in the test manual. Testing lasted approximately one hour, with a suitable number of breaks to minimize the effects of fatigue and frustration.”*

**Reviewer comments:**

April 2018 to June 2020 was this just recruitment or when the whole study took place? If it was a two-year study, there needs to be more in the procedures.

**Reply:**

Thank you for the question. This study recruited participants from April 2018 to June 2020.

**Reviewer comments:**

Line 147-149: why is the DBRS questionnaire important here?

**Reply:**

Thank you for the question. All of participants were asked to complete the Disruptive Behavior Rating Scales (DBRS) questionnaire by their parents for investigating ADHD tendency. Because the children with strabismus have shown an increased risk (OR=2.13, 95% CI=1.52–2.99) for attention deficit hyperactivity disorder (ADHD) (Reimelt C et al., 2021). Considering the relevance of childhood strabismus to ADHD-related symptoms, whether the BOT-2 SF is effective in testing the motor competency of preschool children with strabismus is worthy of further discussion.

**Results:**

**Reviewer comments:**

I would recommend putting the percentages near each classification, so it makes it easier for the reader.

**Reply:**

Thank you for the suggestion. This has been corrected (Page 14, lines 194−196):*” On the BOT-2 CF, 15% in the “Fine Manual Control” composite, 70% in the "Manual Coordination" composite, 32.5% in the "Body Coordination" composite, and 5% in the "Strength and Agility" composite of preschool children with strabismus …”*

**Reviewer comments:**

Lines 197-198: Why are these results important? This is not part of your purpose statement.

**Reply:**

Thank you for the question. Because the children with strabismus have shown an increased risk (OR=2.13, 95% CI=1.52–2.99) for attention deficit hyperactivity disorder (ADHD) (Reimelt C et al., 2021). And Mancini and colleagues (2020) reported that the BOT-2 SF overestimates a child's motor proficiency relative to the BOT-2 CF in school-age children with ADHD. Therefore, we would like to confirm the results of applying BOT-SF to preschool children with strabismus. The purpose has been amended on (Page 9, lines 85−86): “*Finally, possible confounding factors, such as ADHD tendencies and stereopsis were also examined.”*

**Discussion**

**Reviewer comments:**

Authors mention esotropia in the third paragraph, however, it seems a bit unclear what Esotropia is because it has not been mentioned up until this point. If it is a necessary component of Strabismus then I would recommend putting this into your introduction.

**Reply:**

Thank you for the suggestion. We have rewritten the Introduction to clearly state the strabismus (Page 4, lines 43−44): *“Strabismus is defined as a deviation of the eye from perfect ocular alignment and can be further divided into esotropia and exotropia, or less commonly, hypertropia and hypotropia (1).”*

**Reviewer comments:**

In the 6^th^ paragraph starting with line 278-292 authors talk about the finding of the DBRS questionnaire. If this is something that will be used in the study, it needs to be mentioned in the methods and the questionnaire should be described. Also, this should be part of the research question/purpose.

**Reply:**

Thank you for the suggestion. This has been amended on (Page 12, lines 146−157): “*The Disruptive Behavior Rating Scale (DBRS) questionnaire is a brief (26-item) rating scale used to assess inattention, hyperactivity–impulsivity, and oppositional defiant behavior in preschool-aged children (26). The DBRS assesses 18 symptoms of ADHD that indicate inattention and hyperactivity–impulsivity, but also contains 8 additional items to assess oppositional defiant disorder (ODD) symptoms. The cut-off score of inattention is 15 points for boys and 12 points for girls. The cut-off score of hyperactivity–impulsivity is 17 points for boys and 13 points for girls. The use of the DBRS as a measure of disruptive behavior disorder symptoms in preschool-aged children showed strong internal consistency and evidence of convergent/divergent and discriminative validity (27). This study used 18 items of the DBRS to investigate ADHD tendencies of participants.”*; (Page 8−9, lines 79−82): *“Considering the relevance of childhood strabismus to ADHD-related symptoms, whether the BOT-2 SF is effective in testing the motor competency of preschool children with strabismus is worthy of further discussion.”*; (Page 9, lines 85−86): *“Finally, possible confounding factors, such as ADHD tendencies and stereopsis were also examined.”*

**Reviewer comments:**

In paragraph 8, authors speak about person’s correlations and the results. Were correlations used to analyze these data? If so, this needs to be mentioned in the data analysis section as well.

**Reply:**

Thank you for the suggestion. This has been amended on (Page 13−14, lines 188−190): “*Pearson's product-moment coefficients were used to assess whether the 14 items included in the BOT-2 SF test were indeed representative of the respective domains in the BOT-2 CF test (31).”*
